# Effects of Single-Nucleotide Polymorphisms on the Estimated Breeding Values for Feet in Holstein-Friesian Cows in Hungary

**DOI:** 10.3390/ani16091299

**Published:** 2026-04-23

**Authors:** Attila Zsolnai, László Bognár, Szabolcs Albin Bene, Zsolt Jenő Kőrösi, László Rózsa, Ferenc Szabó, István Anton

**Affiliations:** 1 Institute of Animal Husbandry Sciences, Hungarian University of Agriculture and Life Sciences, Guba Sándor utca 40, H-7400 Kaposvár, Hungary; 2 National Association of Hungarian Holstein-Friesian Breeders, Lőportár utca 16, H-1134 Budapest, Hungary; bognar@holstein.hu (L.B.); korosi@holstein.hu (Z.J.K.); 3 Institute of Animal Husbandry Sciences, Hungarian University of Agriculture and Life Sciences, Deák Ferenc utca 16, H-8360 Keszthely, Hungary; bene.szabolcs.albin@uni-mate.hu (S.A.B.); rozsa.laszlo@uni-mate.hu (L.R.); anton.istvan@uni-mate.hu (I.A.); 4 Albert Kázmér Faculty of Mosonmagyaróvár, Széchenyi István University, Vár tér 2, H-9200 Mosonmagyaróvár, Hungary; szabo.ferenc@sze.hu

**Keywords:** cattle, genomic selection, genetic marker, feet, estimated breeding value

## Abstract

The aim of this study was to identify SNPs in the cattle genome associated with estimated breeding values of feet (EBV_feet_) in Holstein-Friesian (HF) cows in Hungary. Foot health is of major importance in dairy cattle industry whereas claw disorders are leading to lameness and thus result in low fertility rates and productivity. Genotyping was performed using the EuroG_MDv4 microarray platform. The final database comprised 2963 animals and 59,151 SNPs. EBV_feet_ values have been divided into high and low groups. All calculations regarding the genetic differentiation (genome-wide and locus-specific) between high- and low-value groups for EBV_feet_, linear regression, and haplotype association tests have been performed with the SNP and Variation Suite software. Thirty-nine SNPs associated with EBV_feet_ were determined on BTAs 3, 7, 8, 15, 21, and X. The maximum values of the identified SNPs were 0.22 for F_st_marker_, 23.1 for the −log10(p) of the linear regression, and 26.3 for the −log10(p) of the haplotype association tests on BTA 3. The closest genes to SNPs associated with estimated breeding values for feet (EBV_feet_) are mainly associated with tissue structure, immune response, metabolism, growth, development, transport and signaling. Our results could add additional information to the genetic programs focusing on the improvement of foot health in HF cattle.

## Simple Summary

Through a genome-wide association study, genomic regions have been identified that affect feet traits of Holstein-Friesian cows in Hungary. The closest genes to SNPs associated with estimated breeding values for feet (EBV_feet_) are mainly associated with tissue structure, immune response, metabolism, growth and development, transport, and signaling. Thirty-nine SNPs associated with EBV_feet_ were identified on chromosomes 3, 7, 8, 15, 21, and X. Our results could additional information to the genetic programs focused on the improving foot health in Holstein-Friesian cattle.

## 1. Introduction

Beyond other traits [1], foot health is of critical importance in the dairy cattle industry. The claw disorders lead to lameness were associated with low fertility rates and productivity in Holstein-Friesian (HF) cows in Spain, and have also been linked to decreased longevity [2].

Tarsani et al. identified multiple genes (*ALOXE3*, *ALOX12B*, *ALOX12E*, *ALOX15*, *ALOX12*, *CMKLR1*, *C1QBP*, and *ARRB2*) associated with digital dermatitis in HF cows at different lactation stages [3]. A multi-trait GWAS in Chinese Holstein cattle revealed 54 SNPs near to several candidate genes associated with body conformation traits, and feet/legs (*GNAQ*, *SAMHD1*, and *SOGA1*) traits [4]. Multiple markers were identified for different hoof health traits in North American HF cattle, including digital and interdigital dermatitis. The identified genomic regions were associated with some previously described loci for health and production traits [5]. Genetic and genomic analyses in American and Australian Angus cattle revealed several candidate genes (*FGF12* and *GDF5*) for foot score traits, which had been previously associated with bone structure [6]. When analyzing the genetic background of claw horn lesions in HF cattle in Scotland, some candidate genomic regions were identified as associated with susceptibility to sole hemorrhage, sole ulcers, and white line disease on BTA3 and BTA18 [7].

Associations between copy number variants and hoof health traits have been investigated in HF cattle employing estimated breeding values (EBVs). Most of the identified genes (*SCART1*, *NRXN2*, *KIF26A*, *GPHN*, and *OR7A17*) were associated with different metabolic disorders [8]. A two-stage GWAS in dairy cattle identified 33 SNPs located on BTA7 between the genes *RIOK2* and *RGMB* that were associated with the total number of hoof disorders [9]. Indian Vrindavani cows with AG, TT, and GG genotypes at loci rs41632254, rs41603160, and rs41636945, respectively, were more resistant to lameness [10]. Ten significant SNPs in HF cattle (false discovery rate ≤ 0.05) were associated with sole ulcer (located on BTA8, BTA10, BTA11, BTA18, and BTA22) and three suggestive SNPs (false discovery rate ≤ 0.20) were connected to double sole, interdigital hyperplasia and sole ulcer (located on BTA13, BTA14, and BTA17) [11]. Multiple candidate genes have been identified as associated with feet and leg conformation traits in Chinese HF cattle, including *ADIPOR2*, *INPP4A*, *DNMT3A*, *ALDH1A2*, *PCDH7*, *XKR4*, and *CADPS* [12].

Considering the importance of foot health in the dairy industry, this study aimed to identify SNPs associated with the EBVs for feet (EBV_feet_) traits in HF cows in Hungary. For the SNP identification, common top hits of three different algorithms were combined; (i). F_st_ approach: if a specific SNP shows an exceptionally high F_st_, it suggests that natural selection or a specific trait is driving that an allele to higher frequency in one group. (ii). Linear regression method: genotypes are independent variables and the trait is the dependent one. For each SNP, we test the null hypothesis that the effect size is zero. A probability value is assigned to SNPs, and small *p*-value suggests that the association of an SNP with the trait is very unlikely to have happened by chance. (iii). Haplotype association test: after identification and phasing of haplotype blocks, probability values calculated on these blocks are giving information on association, like in linear regression.

## 2. Materials and Methods

The phenotypic and genotypic records of animals were furnished by the National Association of Hungarian Holstein-Friesian Breeders which supports and coordinates the genomic selection of HF cattle in Hungary. The five standard traits recorded for legs and feet are: rear leg set, rear leg rear view, foot angle, foreleg set, and locomotion. These traits are based on the most recent (2023) recommendation of the International Committee for Animal Recording (ICAR) [13] and scored once around the 100th day of the first lactation. The score is recorded on a scale of 1–9 with 5 being the best score for leg and foot positions, and 9 being the best score for locomotion. The traits are then summarized in an index and an EBV for this index (EBV_feet_) was calculated as described by Bognar et al. [14], where the prior distribution of the Bayesian model was a mixture distribution. The polygenic effect relies on a pedigree-based relationship matrix, its variance structure is modeled as a multivariate normal distribution.

All individuals were genotyped using the EuroG_MDv4 microarray (Eurogenomics, Amsterdam, The Netherlands), which included 67,227 SNPs. SNPs with GeneTrain score above 0.25, call rate above 0.95 and minor allele frequency above 0.05 were retained in the dataset. Only samples with a call rate higher than 0.95 were included in the study. The number of the kept SNPs were 66,488, 65,690 and 59,151, respectively. Four duplicated samples were excluded after identity by state test. Succeeding the quality inspection of samples, our database comprised 2963 animals and 59,151 SNPs.

EBV_feet_ values have been divided into high and low groups. One possible approach to select the amount of the samples for analysis is to determine where samples are falling into 2 sigma distribution, then select for the remaining animals from the tails. This means 2.5 percentages from each tail. In our case we have chosen to select more animals, so we lowered sigma value to 1.4, which has given us 8-8 percent of animals from the tails of the distribution. Eight percent give 237 animals in each EBV_feet_ class. The genetic differentiation value between the high and low groups was 0.008. The cutoff values were: EBV_feet_high_ > 0.91, EBV_feet_low_ < −0.2.

Population differentiation was estimated based on genome-wide F_st_ and locus-specific F_st_ (F_st_marker_). All calculations [15] regarding genetic differentiation of high and low groups, genetic differentiation of SNPs [F_st_marker_], linear regression [16], and haplotype association tests [17] have been performed with the SNP and Variation Suite (SVS) software (version 8.8.1; Golden Helix, Bozeman, MT, USA). The SNP and Variation Suite addresses rare haplotypes through its modules that include collapsing methods and kernel-based tests to aggregate rare haplotype information.

The study utilized a chi-squared test applied to haplotypes within a sliding five-marker window. A 5-SNP window spans enough markers to capture regional LD patterns [18]. To construct these haplotypes, we employed an expectation-maximization (EM) algorithm with parameters; maximum iterations = 50, convergence tolerance = 0.0001. Thresholds for EBV_feet_ associations were set as: F_st_marker_ ≥ 0.07; −log_10_(p) from the linear regression ≥ 11; and −log_10_(p) from the haplotype test ≥ 11. False discovery rates (FDR) of the SNPs were required to be below 0.02. The F_st_marker_ threshold was chosen to achieve at minimum a moderate differentiation between the two EBV_feet_ groups per SNP.

In case of markers BTB-00549060 (BTA7), ARS-BFGL-NGS-106172 (BTA15), and ARS-BFGL-NGS-75316 (BTA21) the closest genes were located beyond 1 MB. Distances are reported at Supplementary Table S1. The ARS-UCD1.2 B. taurus genome assembly was used as reference.

## 3. Results

Means and standard deviations for EBV_feet_low_ were −0.50 ± 0.26, minimum and maximum values were −1.58 and −0.2, respectively. Means and standard deviations for EBV_feet_high_ were 1.12 ± 0.16, minimum and maximum values were 0.91 and 1.73, respectively. Number of animals was 237 in both the high and low groups.

**Figure 1:**
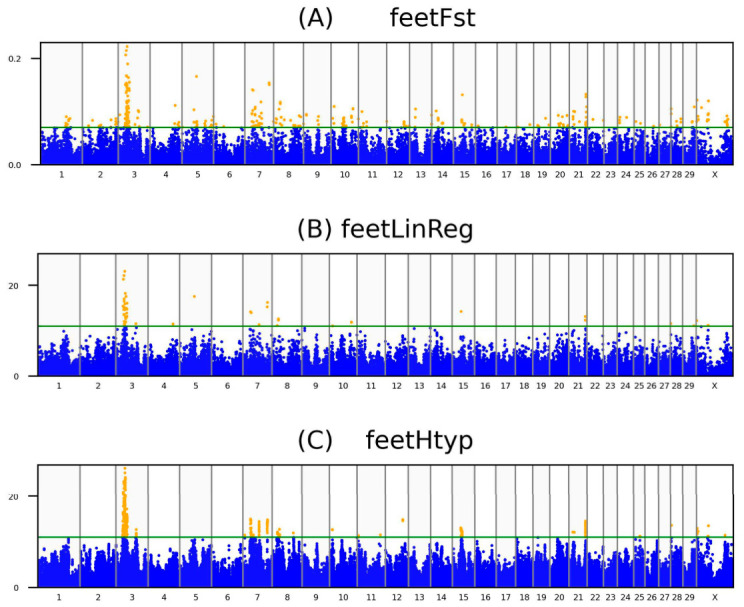
The Manhattan plots represent the applied analytical methods for the feet trait (EBV_feet_). The rows show F_st_marker_ outcomes (**A**), linear regression (**B**), and haplotype correlations (**C**), respectively. The orange dots, the SNPs, positioned above the green lines (thresholds) represent those SNPs which were used for identifying the common hits.

The analysis identified 39 significant SNPs with observed FDR values ranging from 1.9 × 10^−21^ to 1.0 × 10^−4^. These SNPs associated with EBV_feet_ were identified on BTAs 3, 7, 8, 15, 21, and X (Supplementary Table S1). The maximum values of the identified SNPs were 0.22 for F_st_marker_, 23.1 for the −log_10_(p) of the linear regression, and 26.3 for the −log_10_(p) of the haplotype association tests on BTA 3. Table 1 provides an overview of the properties of genes nearest to the reported SNPs, while their full nomenclature is detailed in Supplementary Table S1. For F_st_marker_ values, −log_10_(p) scores of linear and haplotype regressions, regression beta coefficients, and false discovery rates, refer to Supplementary Table S2. Additionally, the allele frequencies are in Supplementary Table S3. Manhattan plots are on Figure 1, while the Q-Q plots corresponding to Figure 1B,C are provided in Supplementary Figure S1.

## 4. Discussion

The aim of this study was to identify SNPs in the cattle genome associated with EBV_feet_. Three algorithms were employed to find the most important candidate genes.

The reported markers were located in the vicinity to candidate genes, with a median distance of 260 kilobases; this increased to 338 kilobases when including the markers identified on BTA7, 15, and 21. While the average distance of linkage in cattle typically ranges from 50 to 100 kilobase [42] and generally decays exponentially as distance increases, long-range linkage disequilibrium regions have been documented. These regions, which span 1–5 Mb between haplotype blocks [43], have been previously documented in French cattle breeds. Therefore, after sequencing, it is necessary to analyze the linkage disequilibrium between the reported SNPs and variants in the *PRR16*, *TECTA*, *PIK3C2A*, and *SETD3* genes (Supplementary Table S1), which are located over 1 Mb apart. Furthermore, neighboring structural variants in vicinity of the reported SNPs remained unexplored in the current study.

The closest genes to SNPs associated with EBV_feet_ are mainly associated with tissue structure, immune response, metabolism, growth, development, transport and signaling (Table 2). The identified genes emphasize the importance of the immune system, energy balance and cellular signaling processes necessary for the growth and integrity of feet tissues.

The *SLF* gene, previously reported for its connection to udder traits [35], was associated with the EBV_feet_ in our analyses. New associations with EBV_feet_ were found for *SLC22A15* and *SLC30A7*, both on BTA3 where the strongest association signals were observable by the applied algorithms.

### 4.1. The Closest Genes to SNPs Associated with EBV_feet_

#### 4.1.1. Tissue Structure

While TECTA is associated with hearing [37] through its activity in the extracellular matrix [44], the integrity of the extracellular matrix is equally vital in the connective tissues of the foot, such as the dermis, ligaments, and tendons, which provide structural support. Proteins like SLC22A15, linked to muscular development [20], and PIK3C2A, near a muscle weight QTL [45], are indirectly relevant as muscle function and limb structure influence locomotion and the mechanical forces acting on the foot.

#### 4.1.2. Immune Response

The bovine foot is constantly exposed to environmental pathogens [46]. Proteins like CD101 [19] and ST7L [25], involved in cell status, and FAM19A3, which regulates immune cell polarization [24], are highly relevant to the foot’s ability to respond effectively to infections like foot rot and digital dermatitis and manage inflammation.

#### 4.1.3. Metabolism

Maintaining a healthy hoof and digital cushion requires proper metabolism and nutrient supply [47]. DENND4C, associated with intramuscular fat [36], and SLC30A7, a zinc transporter involved in fat metabolism [32] and used in treating ketosis [31], highlight the importance of lipid and mineral metabolism for foot health. Zinc, in particular, is crucial for keratinization, the process of hoof horn formation [47]. The association of AMPD2 with skeletal muscle reaction to cold stress [26] suggests a broader role in tissue metabolic responses that could extend to the peripheral areas, such as the limbs.

#### 4.1.4. Growth, Development, and Differentiation

TCOF1 is involved in craniofacial development [21], which shares underlying processes with bone and cartilage formation in the foot. CSNK1A1 has a key role in embryonic development [34] and various cellular processes, potentially including differentiation in hoof growth. RPA4 maintains genomic integrity [41], which is fundamental for the health and function of all actively dividing cells, including those in the hoof matrix.

#### 4.1.5. Transport and Signaling

Proteins like CYB561D1, a transmembrane transporter [27], and SLC22A15, a transporter [48], underscore the fundamental need for efficient transport of molecules in and out of the cells of the foot for nutrient uptake, waste removal, and signaling. CSNK1A1, a protein kinase, is involved in phosphorylation [49], a key regulatory mechanism in numerous cellular signaling pathways that control processes from cell growth to inflammatory responses.

#### 4.1.6. Indirect Physiological Links

While proteins like SYT6 and RNPC3 are directly related to reproduction [23] and endocrine function [30], the overall physiological state of the animal, influenced by the reproduction and endocrine systems, can indirectly impact foot health. For example, metabolic stresses like ketosis can predispose cattle to lameness [50]

## 5. Conclusions

GWAS results published so far suggest that foot health and resistance to claw disorders can potentially be used by genomic selection. Genes related to development and differentiation (*TCOF1*, *CSNK1A1*, *RPA4*), transport and signaling (*CYB561D1*, *SLC22A15*, *CSNK1A1*), tissue structure (*TECTA*, *SLC22A15*, *PIK3C2A*), immune response (*CD101*, *ST7L*, *FAM19A3*) and metabolism (*DENND4C*, *SLC30A7*, *AMPD2*) appear to influence EBVs for feet. Our results could add additional information to the genetic projects focusing on the improvement of foot health in HF cattle. Further studies based on more dense marker maps could remarkably improve EBV_feet_ in dairy cattle.

## Figures and Tables

**Table 1:** List of the closest genes to SNPs associated with EBV_feet_, their properties and species investigated in the cited literature. The order of SNPs follows the position they occupy on the chromosome.

Gene	Properties	Species
*CD101*	Its expression could be used as an indicator of the status and efficacy of therapies in different cancers [19].	Human
*SLC22A15*	Under selection for muscular development in the Large White [20].	Pig
*TCOF1*	Involved in craniofacial abnormalities during prenatal development [21] and breast cancer [22].	Human
*SYT6*	Has a role in the regulation of fertility in giant pandas [23].	Panda
*FAM19A3*	Regulates microglia/macrophage polarization and improved the symptoms of cerebral ischemia [24].	Mouse
*ST7L*	Its effect on severe sepsis has been validated [25].	Human
*AMPD2*	Associated with the reaction of skeletal muscle to cold stress in indigenous Chinese Min breed [26].	Pig
*CYB561D1*	Involved in transmembrane transport [27], associated with autism in humans [28]. Identified as a tumor suppressor in mice [29].	Human, Mouse
*RNPC3*	Associated with hypopituitarism and primary ovarian insufficiency in female mice [30].	Mouse
*SLC30A7*	This zinc transporter was used in treating dairy cows with clinical ketosis [31], and associated with improved fat metabolism and cardiorespiratory functions, which can help Tibetan pigs in their high-altitude adaptation [32].	Cattle, Pig
*PRR16*	Associated with the flesh color of Duroc × Landrace × Large White crossbred [33].	Pig
*CSNK1A1*	Has a key role in the embryonic development [34].	Mouse
*SLF1*	Associated with udder traits in Chinese Holstein [35].	Cattle
*DENND4C*	Associated with intramuscular fat [36].	Rabbit
*TECTA*	It was associated with hearing loss in humans [37].	Human
*PIK3C2A*	Participates in amyloid pathology in mice [38].	Mouse
*SETD3*	Has a role in muscle memory via methylation state in humans [39]. Its deficiency decreased litter sizes in mice [40].	Human, Mouse
*RPA4*	Supports processes maintaining the genomic integrity of the cell [41].	Human

**Table 2:** The nearest genes to SNPs associated with EBV_feet_ grouped by biological functions Genes marked with the same color denote involvements in different biological functions.

	Tissue Structure	Immune Response	Metabolism	Growth, Development, and Differentiation	Transport and Signaling	Indirect Physiological Links
EBV_feet_	*TECTA*, *SLC22A15*, *PIK3C2A*, *PRR16*, *SETD3*	*CD101*, *ST7L*, *FAM19A3*, *CSNK1A1*	*DENND4C*, *SLC30A7*, *AMPD2*, *PRR16*, *SETD3*	*TCOF1*, *CSNK1A1*, *RPA4*	*CYB561D1*, *SLC22A15*, *CSNK1A1*	*SYT6*, *RNPC3*, *SLF1*

## Data Availability

The raw dataset presented in this article is not readily available because the data are part of ongoing studies and are owned by the National Association of Hungarian Holstein-Friesian Breeders. Requests to access the datasets should be directed to the second author.

## Supplementary Materials

The following supporting information can be downloaded at: https://www.mdpi.com/article/10.3390/ani16091299/s1, Table S1: The names of the markers associated with feet EBV, their genomic positions (B. taurus genome build ARS-UCD1.2), and the closest genes (symbol); Table S2: SNP markers associated with EBV_feet_. The F_st_marker_ columns are the genetic differentiation of the markers, Linear Regression −log10(p) columns are the transformed *p* values calculated via linear regression. Regression Beta is the effect size on a particular trait revealing both the strength and direction of the association between a single nucleotide polymorphism (SNP) and the phenotype of interest. Negative values are signed by red horizontal bars, positive values are signed by green bars. Htype −log10(p) columns are the transformed *p* values calculated via haplotype regression. False Discovery Rate (FDR) values are displayed in case of linear (LinReg FDR) and haplotype regression (HtypeFDR); Table S3: SNP markers associated with EBV_feet_. The nucleotide of minor and major alleles are presented together with their regression beta values. Negative values of regression beta are signed by red horizontal bars, positive values are signed by green bars. A positive beta means the minor allele—which was the test allele in linear regression—increases the trait’s value, a negative beta means the test allele decreases the trait’s value; Figure S1: Q-Q plots of linear regression (B) and haplotype association test (C). The corresponding Manhattan plots are in Figure 1B and Figure 1C, respectively.

## References

[1] Panigrahi M., Rajawat D., Nayak S.S., Jain K., Vaidhya A., Prakash R., Sharma A., Parida S., Bhushan B., Dutt T. (2024). Genomic insights into key genes and QTLs involved in cattle reproduction. Gene.

[2] Charfeddine N., Perez-Cabal M.A. (2017). Effect of claw disorders on milk production, fertility, and longevity, and their economic impact in Spanish Holstein cows. J. Dairy Sci..

[3] Tarsani E., Li B., Anagnostopoulos A., Barden M., Griffiths B.E., Bedford C., Coffey M., Psifidi A., Oikonomou G., Banos G. (2025). Genome-wide association studies of dairy cattle resistance to digital dermatitis recorded at four distinct lactation stages. Sci. Rep..

[4] Li S., Ge F., Chen L., Liu Y., Chen Y., Ma Y. (2024). Genome-wide association analysis of body conformation traits in Chinese Holstein Cattle. BMC Genom..

[5] Sousa L.P.B.J., Pinto L.F.B., Cruz V.A.R., Oliveira G.A., Rojas de Oliveira H., Chud T.S., Pedrosa V.B., Miglior F., Schenkel F.S., Brito L.F. (2024). Genome-wide association and functional genomic analyses for various hoof health traits in North American Holstein cattle. J. Dairy Sci..

[6] Alvarenga A.B., Retallick K.J., Garcia A., Miller S.P., Byrne A., Oliveira H.R., Brito L.F. (2023). Across-country genetic and genomic analyses of foot score traits in American and Australian Angus cattle. Genet. Sel. Evol..

[7] Li B., Barden M., Kapsona V., Sanchez-Molano E., Anagnostopoulos A., Griffiths B.E., Bedford C., Dai X., Coffey M., Psifidi A. (2023). Single-step genome-wide association analyses of claw horn lesions in Holstein cattle using linear and threshold models. Genet. Sel. Evol..

[8] Butty A.M., Chud T.C.S., Cardoso D.F., Lopes L.S.F., Miglior F., Schenkel F.S., Canovas A., Hafliger I.M., Drogemuller C., Stothard P. (2021). Genome-wide association study between copy number variants and hoof health traits in Holstein dairy cattle. J. Dairy Sci..

[9] Suchocki T., Egger-Danner C., Schwarzenbacher H., Szyda J. (2020). Two-stage genome-wide association study for the identification of causal variants underlying hoof disorders in cattle. J. Dairy Sci..

[10] Prakash C., Gaur G.K., D R P., Sahoo N.R. (2020). Distribution analysis of single nucleotide polymorphisms related to feet and legs and their association with lameness in Vrindavani cattle. Trop. Anim. Health Prod..

[11] van der Spek D., van Arendonk J.A., Bovenhuis H. (2015). Genome-wide association study for claw disorders and trimming status in dairy cattle. J. Dairy Sci..

[12] Abdalla I.M., Lu X., Nazar M., Arbab A.A.I., Xu T., Yousif M.H., Mao Y., Yang Z. (2021). Genome-Wide Association Study Identifies Candidate Genes Associated with Feet and Leg Conformation Traits in Chinese Holstein Cattle. Animals.

[13] Appendix 1 of Section 5 of the ICAR Guidelines—The Standard Trait Definition for Dairy Cattle. https://www.icar.org/Guidelines/05-Conformation-recording-Appendix-1.pdf.

[14] Bognar L., Korosi Z.J., Bene S.A., Szabo F., Anton I., Zsolnai A. (2024). Simultaneous Effects of Single-Nucleotide Polymorphisms on the Estimated Breeding Value of Milk, Fat, and Protein Yield of Holstein Friesian Cows in Hungary. Animals.

[15] Formulas and Theories. https://www.goldenhelix.com/docs/SVS/latest/svsmanual/formulas_theories.html.

[16] Vilhjalmsson B.J. Mixmogam [Internet]. https://github.com/bvilhjal/mixmogam.

[17] Excoffier L., Slatkin M. (1995). Maximum-likelihood estimation of molecular haplotype frequencies in a diploid population. Mol. Biol. Evol..

[18] Braz C.U., Taylor J.F., Bresolin T., Espigolan R., Feitosa F.L.B., Carvalheiro R., Baldi F., de Albuquerque L.G., de Oliveira H.N. (2019). Sliding window haplotype approaches overcome single SNP analysis limitations in identifying genes for meat tenderness in Nelore cattle. BMC Genet..

[19] Zhou J., Wang W., Liang Z., Ni B., He W., Wang D. (2020). Clinical significance of CD38 and CD101 expression in PD-1(+)CD8(+) T cells in patients with epithelial ovarian cancer. Oncol. Lett..

[20] Atrian-Afiani F., Berger B., Draxl C., Solkner J., Meszaros G. (2023). Selective Sweeps in the Austrian Turopolje and Other Commercial Pig Populations. Animals.

[21] Luo M., Yu X. (2025). NBS1 facilitates preribosomal RNA biogenesis. Proc. Natl. Acad. Sci. USA.

[22] He Q., Hu J., Huang H., Wu T., Li W., Ramakrishnan S., Pan Y., Chan K.M., Zhang L., Yang M. (2024). FOSL1 is a key regulator of a super-enhancer driving TCOF1 expression in triple-negative breast cancer. Epigenet. Chromatin.

[23] Guang X., Lan T., Wan Q.H., Huang Y., Li H., Zhang M., Li R., Zhang Z., Lei Y., Zhang L. (2021). Chromosome-scale genomes provide new insights into subspecies divergence and evolutionary characteristics of the giant panda. Sci. Bull..

[24] Shao Y., Deng T., Zhang T., Li P., Wang Y. (2015). FAM19A3, a novel secreted protein, modulates the microglia/macrophage polarization dynamics and ameliorates cerebral ischemia. FEBS Lett..

[25] Wang L., Zhang A., Hu Y., Yang W., Zhong L., Shi J., Wang Z., Tao Q., Liang Q., Yao X. (2024). Landscape of multiple tissues’ gene expression pattern associated with severe sepsis: Genetic insights from Mendelian randomization and trans-omics analysis. Life Sci..

[26] Zhang D., Ma S., Wang L., Ma H., Wang W., Xia J., Liu D. (2022). Min pig skeletal muscle response to cold stress. PLoS ONE.

[27] Asard H., Barbaro R., Trost P., Berczi A. (2013). Cytochromes b561: Ascorbate-mediated trans-membrane electron transport. Antioxid. Redox Signal..

[28] Ning L.F., Yu Y.Q., GuoJi E.T., Kou C.G., Wu Y.H., Shi J.P., Ai L.Z., Yu Q. (2015). Meta-analysis of differentially expressed genes in autism based on gene expression data. Genet. Mol. Res..

[29] Berczi A., Desmet F., Van Doorslaer S., Asard H. (2010). Spectral characterization of the recombinant mouse tumor suppressor 101F6 protein. Eur. Biophys. J..

[30] Akin L., Rizzoti K., Gregory L.C., Corredor B., Le Quesne Stabej P., Williams H., Buonocore F., Mouilleron S., Capra V., McGlacken-Byrne S.M. (2022). Pathogenic variants in RNPC3 are associated with hypopituitarism and primary ovarian insufficiency. Genet. Med..

[31] Chirivi M., Cortes-Beltran D., Gandy J., Contreras G.A. (2025). Oxylipin dynamics in dairy cows during clinical ketosis and after treatment with niacin and flunixin meglumine. JDS Commun..

[32] Yang Y., Yuan H., Yao B., Zhao S., Wang X., Xu L., Zhang L. (2024). Genetic Adaptations of the Tibetan Pig to High-Altitude Hypoxia on the Qinghai-Tibet Plateau. Int. J. Mol. Sci..

[33] Li H., Xu C., Meng F., Yao Z., Fan Z., Yang Y., Meng X., Zhan Y., Sun Y., Ma F. (2022). Genome-Wide Association Studies for Flesh Color and Intramuscular Fat in (Duroc x Landrace x Large White) Crossbred Commercial Pigs. Genes.

[34] Ma Z., Zheng H., Li X., Yu B., Peng H. (2023). Knockdown of Csnk1a1 results in preimplantation developmental arrest in mice. Theriogenology.

[35] Nazar M., Abdalla I.M., Chen Z., Ullah N., Liang Y., Chu S., Xu T., Mao Y., Yang Z., Lu X. (2022). Genome-Wide Association Study for Udder Conformation Traits in Chinese Holstein Cattle. Animals.

[36] Sosa-Madrid B.S., Varona L., Blasco A., Hernandez P., Casto-Rebollo C., Ibanez-Escriche N. (2020). The effect of divergent selection for intramuscular fat on the domestic rabbit genome. Animal.

[37] Alde M., Cantarella G., Zanetti D., Pignataro L., La Mantia I., Maiolino L., Ferlito S., Di Mauro P., Cocuzza S., Lechien J.R. (2023). Autosomal Dominant Non-Syndromic Hearing Loss (DFNA): A Comprehensive Narrative Review. Biomedicines.

[38] Perez Garcia G., Bicak M., Buros J., Haure-Mirande J.V., Perez G.M., Otero-Pagan A., Gama Sosa M.A., De Gasperi R., Sano M., Gage F.H. (2023). Beneficial effects of physical exercise and an orally active mGluR2/3 antagonist pro-drug on neurogenesis and behavior in an Alzheimer’s amyloidosis model. Front. Dement..

[39] Seaborne R.A., Strauss J., Cocks M., Shepherd S., O’Brien T.D., van Someren K.A., Bell P.G., Murgatroyd C., Morton J.P., Stewart C.E. (2018). Human Skeletal Muscle Possesses an Epigenetic Memory of Hypertrophy. Sci. Rep..

[40] Wilkinson A.W., Diep J., Dai S., Liu S., Ooi Y.S., Song D., Li T.M., Horton J.R., Zhang X., Liu C. (2019). SETD3 is an actin histidine methyltransferase that prevents primary dystocia. Nature.

[41] Haring S.J., Humphreys T.D., Wold M.S. (2010). A naturally occurring human RPA subunit homolog does not support DNA replication or cell-cycle progression. Nucleic Acids Res..

[42] Bordbar F., Jensen J., Wadood A.A., Yao Z. (2024). Linkage Disequilibrium Decay in Selected Cattle Breeds. Animals.

[43] El Hou A., Rocha D., Venot E., Blanquet V., Philippe R. (2021). Long-range linkage disequilibrium in French beef cattle breeds. Genet. Sel. Evol..

[44] Legan P.K., Goodyear R.J., Morin M., Mencia A., Pollard H., Olavarrieta L., Korchagina J., Modamio-Hoybjor S., Mayo F., Moreno F. (2014). Three deaf mice: Mouse models for TECTA-based human hereditary deafness reveal domain-specific structural phenotypes in the tectorial membrane. Hum. Mol. Genet..

[45] Chen S., An J., Lian L., Qu L., Zheng J., Xu G., Yang N. (2013). Polymorphisms in AKT3, FIGF, PRKAG3, and TGF-beta genes are associated with myofiber characteristics in chickens. Poult. Sci..

[46] Garvey M. (2022). Lameness in Dairy Cow Herds: Disease Aetiology, Prevention and Management. Dairy.

[47] Langova L., Novotna I., Nemcova P., Machacek M., Havlicek Z., Zemanova M., Chrast V. (2020). Impact of Nutrients on the Hoof Health in Cattle. Animals.

[48] Yee S.W., Buitrago D., Stecula A., Ngo H.X., Chien H.C., Zou L., Koleske M.L., Giacomini K.M. (2020). Deorphaning a solute carrier 22 family member, SLC22A15, through functional genomic studies. FASEB J..

[49] Jiang S., Zhang M., Sun J., Yang X. (2018). Casein kinase 1alpha: Biological mechanisms and theranostic potential. Cell Commun. Signal..

[50] Jukna V., Meškinytė E., Urbonavičius G., Bilskis R., Antanaitis R., Kajokienė L., Juozaitienė V. (2024). Association of Lameness Prevalence and Severity in Early-Lactation Cows with Milk Traits, Metabolic Profile, and Dry Period. Agriculture.

